# Neonatal encephalopathy due to birth asphyxia and trauma: global trends and disparities

**DOI:** 10.3389/fpubh.2025.1583572

**Published:** 2025-09-11

**Authors:** Fengling Du, Long Gu, Junyi Wang, Chan Zhang, Chun Li, Shuai Zhao, Yunchuan Shen, Mei Luo, Yun Yan, Jian Zhou, Wenbin Dong

**Affiliations:** ^1^Department of Neonatology, Children's Medical Center, The Affiliated Hospital, Southwest Medical University, Luzhou, China; ^2^Laboratory of Neurological Diseases and Brain Function, The Affiliated Hospital, Southwest Medical University, Luzhou, China; ^3^Clinical Trial Research Center, The Affiliated Traditional Chinese Medicine Hospital, Southwest Medical University, Luzhou, China; ^4^Department of Neurosurgery, The Affiliated Hospital, Southwest Medical University, Luzhou, China

**Keywords:** neonatal encephalopathy due to birth asphyxia and trauma, asphyxia neonatorum, birth injuries, infant mortality, global health, epidemiology

## Abstract

**Background:**

Neonatal encephalopathy due to birth asphyxia and trauma (NE-BAT) is a leading cause of neonatal mortality and long-term neurodevelopmental disabilities, particularly in low- and middle-income regions.

**Methods:**

Using the Global Burden of Disease (GBD) 2021 dataset, we analyzed global incidence, prevalence, mortality, and disability-adjusted life years (DALYs) of NE-BAT from 1990 to 2021. We examined age-standardized rates, estimated annual percentage changes (EAPC), socio-demographic index (SDI) quintiles, gender differences, and used autoregressive integrated moving average (ARIMA) models for 2031 projections.

**Results:**

From 1990 to 2021, global age-standardized incidence rate declined from 20.22 to 17.16 per 100,000 (EAPC: −0.56), while prevalence increased from 129.73 to 242.03 per 100,000 (EAPC: 2.16). Males had higher incidence (19.93 vs. 14.18 per 100,000) and prevalence (291.46 vs. 191.38 per 100,000) than females. Mortality declined from 13.81 to 9.75 per 100,000 (EAPC: −1.24), and DALYs decreased from 1270.67 to 932.14 per 100,000 (EAPC: −1.12). Low SDI locations had NE-BAT-specific mortality rates 27 times higher than high SDI locations (17.77 vs. 0.66 per 100,000 in 2021). Projections to 2031 suggest continued declines in incidence and DALYs but rising prevalence and mortality rates.

**Conclusion:**

While global burden of NE-BAT has improved through reduced incidence and DALYs, persistent gender and regional disparities, along with rising prevalence rates, pose ongoing challenges. Targeted interventions addressing these disparities, particularly in low SDI locations, are essential to improve neonatal outcomes and reduce health inequalities.

## Introduction

Neonatal encephalopathy due to birth asphyxia and trauma (NE-BAT) remains a critical global health challenge, affecting an estimated 1.2 million newborns annually (1, 2). As the third leading cause of neonatal mortality, NE-BAT accounts for 23% of infant deaths and often results in severe neurological damage and developmental delays due to oxygen deprivation and reduced blood flow to the brain at birth (3). Approximately 60% of moderate to severe neonatal encephalopathy cases are attributable to asphyxia, primarily occurring during labor and delivery, while birth trauma further increases the risk of hypoxic–ischemic encephalopathy (4, 5). Beyond its immediate clinical impact, NE-BAT imposes substantial economic burdens on families and healthcare systems, with recent economic evaluations estimating losses exceeding $80 billion globally among the highest-burden countries, highlighting the critical need for resource allocation and public health prioritization (6).

Despite significant advances in perinatal care, the global burden of NE-BAT shows substantial regional variations in prevalence and outcomes (5). Low- and middle-income countries are disproportionately affected, where limited healthcare resources and infrastructure contribute to higher incidence rates and poorer outcomes (7). While progress has been made in newborn health and survival, achieving the global goal of eliminating preventable newborn deaths and stillbirths by 2030 requires addressing persistent inequalities between countries and regions (8).

The Global Burden of Disease, Injuries, and Risk Factors Study (GBD) provides a comprehensive framework for assessing the impact of NE-BAT at global, national, and subnational levels (9, 10). While previous GBD-based studies have examined general neonatal encephalopathy (6, 11), these studies specifically focus on NE-BAT as a distinct clinical entity combining both asphyxial and traumatic etiologies, which requires targeted prevention and intervention strategies. By analyzing GBD 2021 data on incidence, prevalence, mortality, and disability-adjusted life years (DALYs), this study aims to examine three-decade trends (1990–2021) and project future patterns through 2031 using autoregressive integrated moving average (ARIMA) modeling integrated with frontier analysis. Understanding these patterns, particularly across different socio-demographic index (SDI) quintiles and gender distributions, is crucial for evaluating intervention effectiveness and developing targeted strategies to reduce the global burden of NE-BAT. This study seeks to fill critical gaps in current research by providing comprehensive analysis of long-term trends, future projections, and actionable insights for policymakers and healthcare providers in addressing the evolving challenges of NE-BAT burden globally.

## Methods

### Data source

This study used data from the GBD study to assess the global burden of NE-BAT from 1990 to 2021. The GBD provides extensive data from 204 countries and territories, enabling global, regional, and national analyses of incidence, prevalence, mortality, and DALYs associated with NE-BAT. Age-standardized rates (per 100,000 population) for incidence (ASIR), prevalence (ASPR), mortality (ASMR), and DALYs (ASDR) were calculated using the GBD world population age standard (12). The GBD 2021 data sources and processing methods have been detailed in previous literature (13). This study is a retrospective observational analysis of population-level data, stratified by sex and SDI. 95% uncertainty intervals (UIs) were calculated from 1,000 draws generated by the GBD modeling framework using a comprehensive uncertainty propagation approach that combines uncertainty from multiple sources including data availability, model structure, and parameter estimation. The UIs represent the 25th and 975th values of the ordered distribution of these 1,000 draws.

To quantify temporal trends over the entire study period from 1990 to 2021, estimated annual percentage change (EAPC) was calculated using a linear regression model of the natural logarithm of the age-standardized rates. The year was used as the independent variable, represented by the equation: ln (rate) = *α* + *β* × year, where α denotes the intercept and *β* represents the regression coefficient. The EAPC was then calculated using the formula EAPC = (e^β - 1) × 100%, which converts the regression coefficient to a percentage change per year. This logarithmic transformation linearizes exponential trends and also helps stabilize variance across the time series, ensuring that linear regression assumptions are better satisfied.

### Autoregressive integrated moving average (ARIMA) projections

ARIMA models were used to forecast future trends in NE-BAT burden over the next decade (2022–2031) (14, 15). The ARIMA model incorporates historical data on incidence, mortality, and DALYs to identify underlying patterns in the time series through three key components: autoregressive effects that capture relationships between current and past values, differencing that ensures stationarity by removing trends, and moving average components that account for dependency between observations and residual errors. The model was fitted to epidemiological data from 1990 to 2021 and used to project future trends based on these patterns. ARIMA was selected for its ability to capture temporal dependencies and its robustness in forecasting time series data. ARIMA modeling has demonstrated robust predictive capabilities in recent epidemiological studies, including projections of disease burden trends that inform evidence-based healthcare planning and resource allocation strategies (15).

### Frontier analysis

Frontier analysis was conducted to assess countries’ performance in reducing NE-BAT burden relative to their socioeconomic development level by establishing the optimal performance boundary between SDI and health outcomes. This approach allows for performance benchmarking across countries and territories with varying development levels, where countries and territories positioned closer to the frontier curve demonstrate superior performance relative to their SDI level. Using the SDI, data envelope analysis and LOESS regression were applied to generate and smooth the frontier for age-standardized NE-BAT indicators. This analysis aimed to identify best-performing countries and establish benchmarks for improvement. Detailed methods are available in the supplementary methods.

### Statistical analysis

All statistical analyses were performed using R version 4.3.3. Choropleth maps were generated to visualize the geographical distribution of age-standardized rates and their temporal changes, highlighting regional disparities in NE-BAT burden. The ARIMA prediction was implemented using the forecast package (version 8.23.0) in R.

## Results

### Global incidence and prevalence rates of NE-BAT

Between 1990 and 2021, there was a notable decline in the global burden of NE-BAT. The global ASIR decreased from 20.22 (95% UI: 19.92 to 20.51) per 100,000 in 1990 to 17.16 (95% UI: 16.94 to 17.41) per 100,000 in 2021, with an EAPC of −0.56 (95% CI: −0.62 to −0.51) (Table 1). However, despite this reduction in incidence, the global ASPR of NE-BAT showed a substantial increase over the same period, rising from 129.73 (95% UI: 91.46 to 182.41) per 100,000 in 1990 to 242.03 (95% UI: 210.3 to 273.72) per 100,000 in 2021, with an EAPC of 2.16 (95% CI: 2.12 to 2.21) (Table 1).

**Table 1:** NE-BAT incidence and prevalence by sex and SDI (1990–2021).

Characteristic	1990		2021		EAPC (95% CI) 1990–2021	
	ASIR (95% UI)	ASPR (95% UI)	ASIR (95% UI)	ASPR (95% UI)	Incidence	Prevalence
Global	20.22 (19.92 to 20.51)	129.73 (91.46 to 182.41)	17.16 (16.94 to 17.41)	242.03 (210.3 to 273.72)	−0.56 (−0.62 to −0.51)	2.16 (2.12 to 2.21)
Sex						
Female	16.97 (16.56 to 17.43)	100.73 (70.04 to 144.62)	14.18 (13.88 to 14.51)	191.38 (166.48 to 216.29)	−0.63 (−0.70 to −0.56)	2.26 (2.20 to 2.31)
Male	23.25 (22.89 to 23.66)	158.19 (112.40 to 220.76)	19.93 (19.67 to 20.24)	291.46 (253.38 to 330.70)	−0.52 (−0.56 to −0.47)	2.10 (2.06 to 2.14)
SDI						
High SDI	8.27 (8.11 to 8.45)	175.85 (139.02 to 226.55)	7.18 (7.02 to 7.33)	189.24 (167.11 to 212.90)	−0.35 (−0.39 to −0.31)	0.34 (0.31 to 0.38)
High-middle SDI	14.35 (13.88 to 14.80)	194.24 (141.75 to 267.79)	11.72 (11.42 to 12.04)	276.83 (243.01 to 310.24)	−0.53 (−0.59 to −0.47)	1.48 (1.36 to 1.60)
Middle SDI	19.25 (18.81 to 19.68)	146.77 (98.37 to 217.67)	15.05 (14.77 to 15.33)	293.28 (255.14 to 331.44)	−0.75 (−0.79 to −0.71)	2.35 (2.24 to 2.47)
Low-middle SDI	20.12 (19.64 to 20.61)	53.26 (31.99 to 87.08)	15.14 (14.82 to 15.45)	196.92 (167.89 to 227.41)	−0.95 (−0.97 to −0.94)	4.39 (4.33 to 4.46)
Low SDI	33.85 (33.33 to 34.41)	28.84 (16.02 to 48.82)	25.84 (25.47 to 26.22)	207.92 (169.13 to 250.92)	−0.87 (−1.00 to −0.75)	6.82 (6.49 to 7.15)

NE-BAT, neonatal encephalopathy due to birth asphyxia and trauma; ASIR, age-standardized incidence rates; ASPR, age-standardized prevalence rates; UI, uncertainty interval; CI, confidence interval; EAPC, estimated annual percentage; SDI, socio-demographic index.

The incidence and prevalence rates were higher for males compared to females. The ASIR for males was 19.93 (95% UI: 19.67 to 20.24) per 100,000 in 2021, compared to 14.18 (95% UI: 13.88 to 14.51) per 100,000 for females (Table 1; Supplementary Figure S1A). Similarly, the prevalence in males reached 291.46 (95% UI: 253.38 to 330.7) per 100,000, whereas in females, it was 191.38 (95% UI: 166.48 to 216.29) per 100,000 (Table 1; Supplementary Figure S1B).

ASIR and ASPR trends demonstrated marked disparities across SDI levels. In 2021, high SDI locations had an ASIR of 7.18 per 100,000 (95% UI: 7.02 to 7.33) and an ASPR of 189.24 per 100,000 (95% UI: 167.11 to 212.9), whereas low SDI locations reported an ASIR of 25.84 per 100,000 (95% UI: 25.47 to 26.22) and a much higher ASPR of 207.92 per 100,000 (95% UI: 169.13 to 250.92). The EAPCs for incidence revealed a gradual decrease across SDI quintiles, with high SDI areas showing a smaller decline (−0.35, 95% CI: −0.39 to −0.31) compared to low SDI areas (−0.87, 95% CI: −1 to −0.75) (Table 1; Supplementary Figures S1C–F).

### Global mortality and DALYs rates of NE-BAT

The global ASMR for NE-BAT decreased significantly between 1990 and 2021. In 1990, the ASMR was 13.81 (95% UI: 12.65 to 15.71) per 100,000, but by 2021, this had decreased to 9.75 (95% UI: 8.26 to 11.71) per 100,000, with an EAPC of −1.24 (95% CI: −1.49 to −0.98) (Table 2). The mortality rates were higher for males compared to females. The ASMR for males was 11.07 (95% UI: 9.15 to 13.21) per 100,000 in 2021, compared to 8.34 (95% UI: 6.95 to 9.94) per 100,000 for females (Table 2; Supplementary Figure S2A).

**Table 2:** NE-BAT mortality and DALYs by sex and SDI (1990–2021).

Characteristic	1990		2021		EAPC (95% CI) 1990–2021	
	ASMR (95% UI)	ASDR (95% UI)	ASMR (95% UI)	ASDR (95% UI)	Mortality	DALYs
Global	13.81 (12.65 to 15.71)	1270.67 (1164.59 to 1443.71)	9.75 (8.26 to 11.71)	932.14 (796.29 to 1101.54)	−1.24 (−1.49 to −0.98)	−1.12 (−1.37 to −0.86)
Sex						
Female	11.86 (10.52 to 13.7)	1089.64 (967.93 to 1256.78)	8.34 (6.95 to 9.94)	795.36 (670.51 to 942.27)	−1.28 (−1.41 to −1.15)	−1.16 (−1.28 to −1.04)
Male	15.63 (13.97 to 17.96)	1439.32 (1291.01 to 1657.61)	11.07 (9.15 to 13.21)	1060.17 (889.77 to 1252.58)	−1.20 (−1.33 to −1.07)	−1.08 (−1.20 to −0.96)
SDI						
High SDI	1.60 (1.51 to 1.71)	181.67 (164.79 to 206.75)	0.66 (0.58 to 0.72)	99.37 (86.55 to 112.88)	−2.58 (−2.73 to −2.42)	−1.72 (−1.95 to −1.49)
High-middle SDI	6.79 (6.10 to 7.66)	653.58 (581.36 to 731.17)	1.09 (0.93 to 1.27)	157.82 (136.66 to 182.89)	−6.16 (−6.42 to −5.90)	−4.92 (−5.18 to −4.66)
Middle SDI	11.50 (10.14 to 12.89)	1066.96 (937.37 to 1190.50)	4.06 (3.42 to 4.86)	429.93 (369.87 to 501.71)	−3.47 (−3.75 to −3.20)	−3.06 (−3.33 to −2.79)
Low-middle SDI	17.86 (15.66 to 21.50)	1618.30 (1424.28 to 1950.85)	12.07 (9.90 to 14.49)	1130.55 (942.77 to 1350.76)	−1.28 (−1.51 to −1.05)	−1.18 (−1.41 to −0.95)
Low SDI	23.94 (21.02 to 28.73)	2159.51 (1895.70 to 2591.41)	17.77 (14.77 to 21.58)	1653.75 (1388.53 to 1994.40)	−0.80 (−1.09 to −0.51)	−0.71 (−1.00 to −0.43)

NE-BAT, neonatal encephalopathy due to birth asphyxia and trauma; ASMR, age-standardized mortality rates; ASDR, age-standardized DALYs rates; UI, uncertainty interval; CI, confidence interval; EAPC, estimated annual percentage; SDI, socio-demographic index.

DALYs which measure the overall disease burden by accounting for both mortality and disability, also declined globally. The age-standardized DALY rate dropped from 1270.67 (95% UI: 1164.59 to 1443.71) per 100,000 in 1990 to 932.14 (95% UI: 796.29 to 1101.54) per 100,000 in 2021, with an EAPC of −1.12 (95% CI: −1.37 to −0.86) (Table 2). Similar to mortality, the DALY rate was higher for males than for females, with males experiencing 1060.17 (95% UI: 889.77 to 1252.58) DALYs per 100,000 in 2021, compared to 795.36 (95% UI: 670.51 to 942.27) for females (Table 2; Supplementary Figure S2B).

For mortality and DALYs, low SDI locations consistently demonstrated the highest burden, with an ASMR of 17.77 per 100,000 (95% UI: 14.77 to 21.58) and ASDR of 1653.75 per 100,000 (95% UI: 1388.53 to 1994.4) in 2021. High SDI locations had significantly lower ASMR and ASDR rates at 0.66 (95% UI: 0.58 to 0.72) and 99.37 per 100,000 (95% UI: 86.55 to 112.88), respectively, with EAPCs reflecting reductions in burden across all SDI levels, particularly in high-middle SDI areas with an EAPC of −6.16 for mortality (95% CI: −6.42 to −5.9) (Table 2; Supplementary Figures S2C–F).

### Geographical distribution of incidence, prevalence, mortality, and DALYs

**Figure 1:**
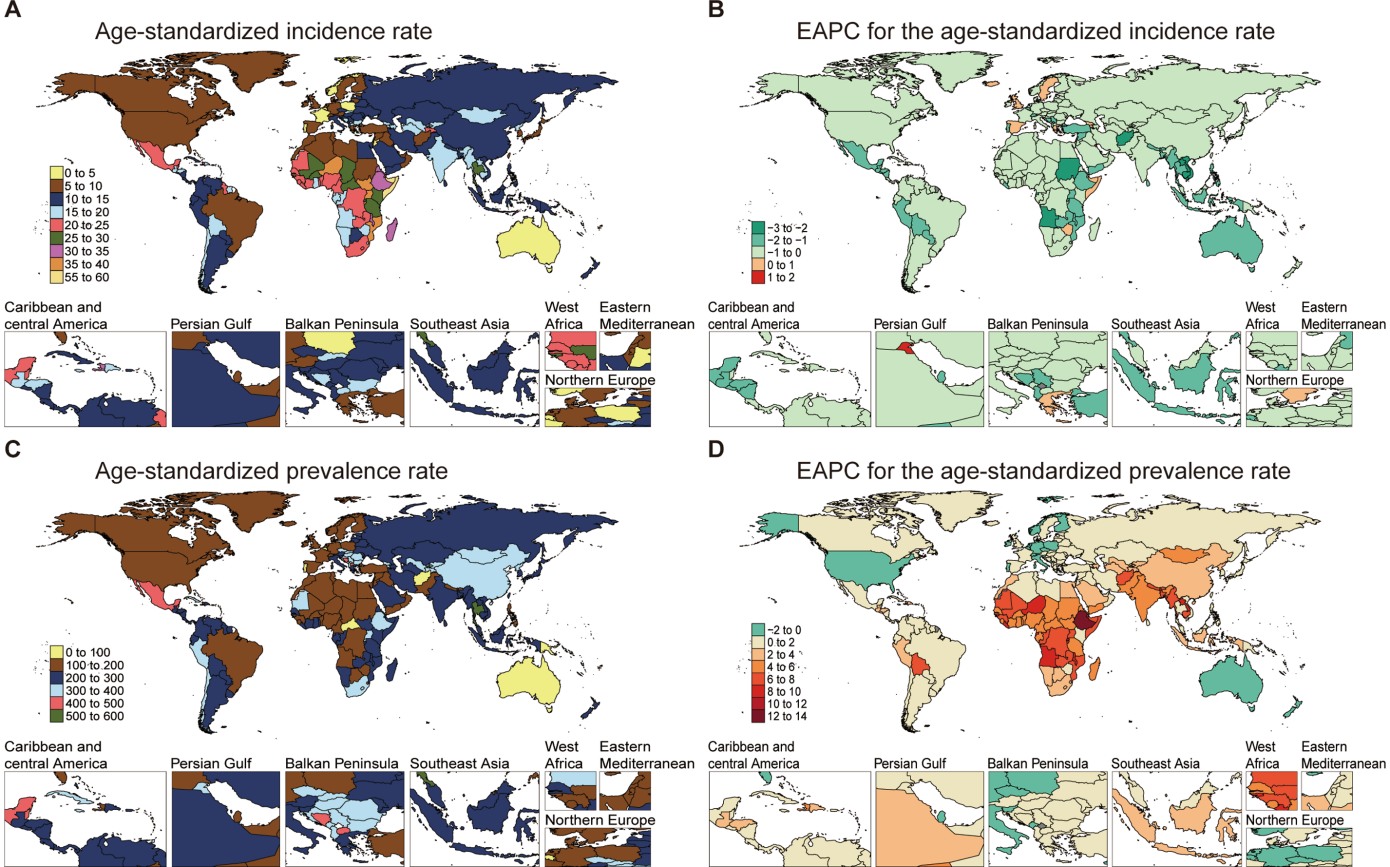
The age-standardized incidence and prevalence rate of NE-BAT in 204 countries and territories. **(A)** The age-standardized incidence rate of NE-BAT in 2021. **(B)** The EAPC of age-standardized incidence rate of NE-BAT from 1990 to 2021. **(C)** The age-standardized prevalence rate of NE-BAT in 2021. **(D)** The EAPC of age-standardized prevalence rate of NE-BAT from 1990 to 2021. NE-BAT, neonatal encephalopathy due to birth asphyxia and trauma; EAPC, estimated annual percentage change.

The ASIR for NE-BAT demonstrated notable geographical disparities in 2021. Somalia recorded the highest ASIR at 56.13 per 100,000 (95% UI: 52.71 to 59.44), whereas Portugal had the lowest ASIR of 3.22 per 100,000 (95% UI: 3.04 to 3.4). Over the 1990 to 2021 period, the EAPC for incidence varied across regions, with Portugal showing a decline (EAPC: -1.76, 95% CI: −1.87 to −1.65), while Somalia showed no obvious changes (EAPC: 0, 95% CI: −0.07 to 0.08) (Figures 1A,B; Supplementary Table S1).

The prevalence rates followed similar regional patterns, with countries like Ethiopia experiencing significant increases in prevalence EAPC (12.25, 95% CI: 11.9 to 12.60). By contrast, Australia showed a declining trend, with an EAPC of −1.04 (95% CI: −1.24 to −0.84). This suggests that the prevalence burden, while widespread, is concentrated in certain regions (Figures 1C,D; Supplementary Table S1).

**Figure 2:**
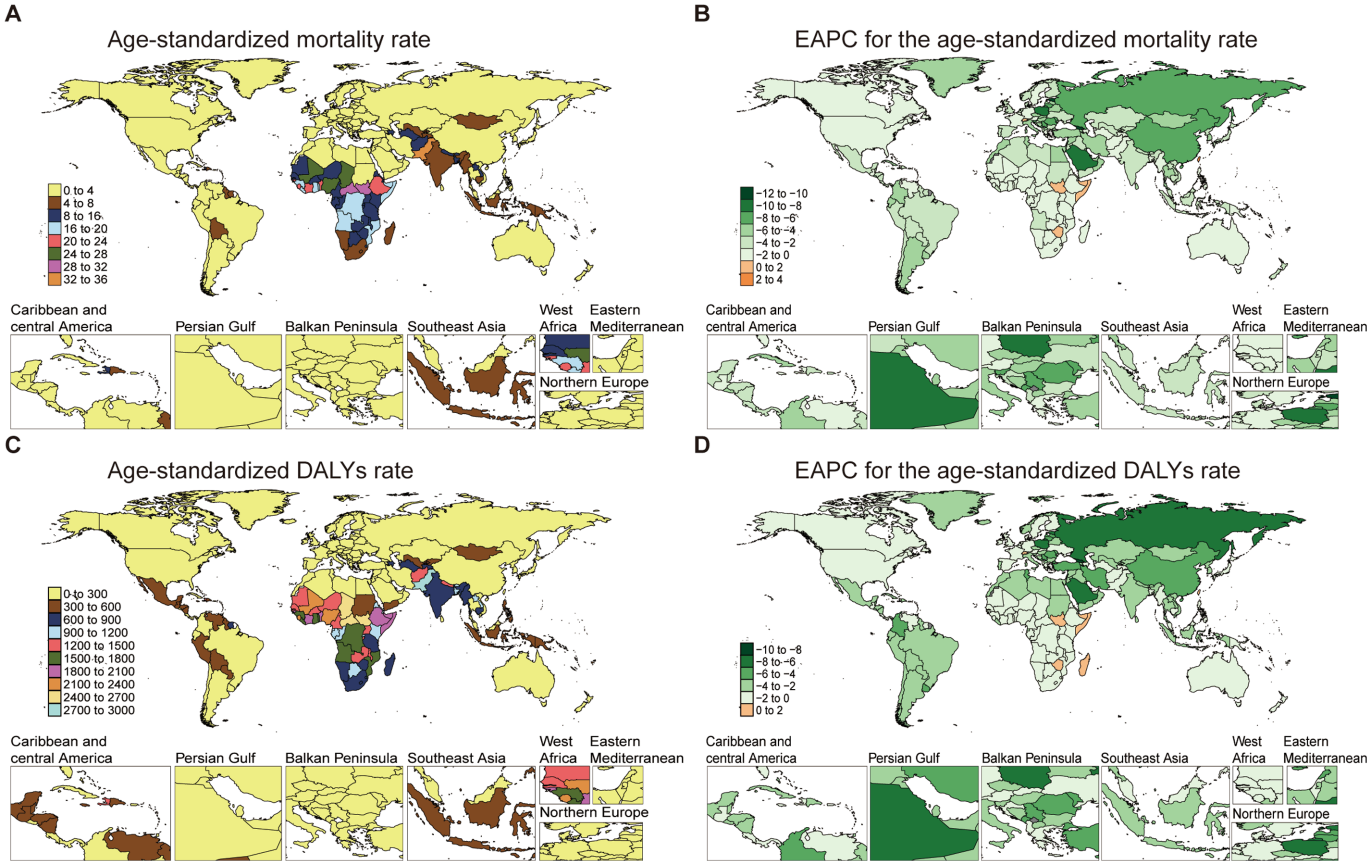
The age-standardized mortality and DALYs rate of NE-BAT in 204 countries and territories. **(A)** The age-standardized mortality rate of neonatal encephalopathy due to birth asphyxia and trauma in 2021. **(B)** The EAPC of age-standardized mortality rate of neonatal encephalopathy due to birth asphyxia and trauma from 1990 to 2021. **(C)** The age-standardized DALYs rate of neonatal encephalopathy due to birth asphyxia and trauma in 2021. **(D)** The EAPC of age-standardized DALYs rate of neonatal encephalopathy due to birth asphyxia and trauma from 1990 to 2021. NE-BAT, neonatal encephalopathy due to birth asphyxia and trauma; EAPC, estimated annual percentage change; DALYs, disability-adjusted life years.

Mortality rates varied widely across countries in 2021. Pakistan presented the highest ASMR at 32.21 per 100,000 (95% UI: 24.64 to 40.19), while Slovenia had the lowest at 0.13 per 100,000 (95% UI: 0.11 to 0.15), representing minimal burden in high-income European settings. EAPC analysis for mortality rates indicated declines in several high-burden regions, with Pakistan’s mortality EAPC at −0.44 (95% CI: −0.61 to −0.27) and Central African Republic at −0.19 (95% CI: −0.31 to −0.07). However, South Sudan showed an increasing trend (EAPC: 1.01, 95% CI: 0.93 to 1.09) (Figures 2A,B; Supplementary Table S2).

The burden of DALYs also exhibited significant variation, paralleling mortality trends. Pakistan’s ASDR remained high at 2927.96 per 100,000 (95% UI: 2245.01 to 3644.70) in 2021, while the lowest rates were observed in Andorra, with an ASDR of 43.76 per 100,000 (95% UI: 32.25 to 56.65). The EAPC for DALYs was highest in Taiwan (Province of China), showing a sustained burden despite overall global improvements, with an EAPC of 1.54 (95% CI: 1.20 to 1.88) over the study period (Figures 2C,D; Supplementary Table S2). Results of cluster analysis based on the EAPC values of NE-BAT-related age-standardized rates of mortality and DALYs from 1990 to 2021 show that Eastern Europe, East Asia, and Central Europe showed significant decreases (Supplementary Figure S3).

### Frontier analysis of NE-BAT prevention and treatment

**Figure 3:**
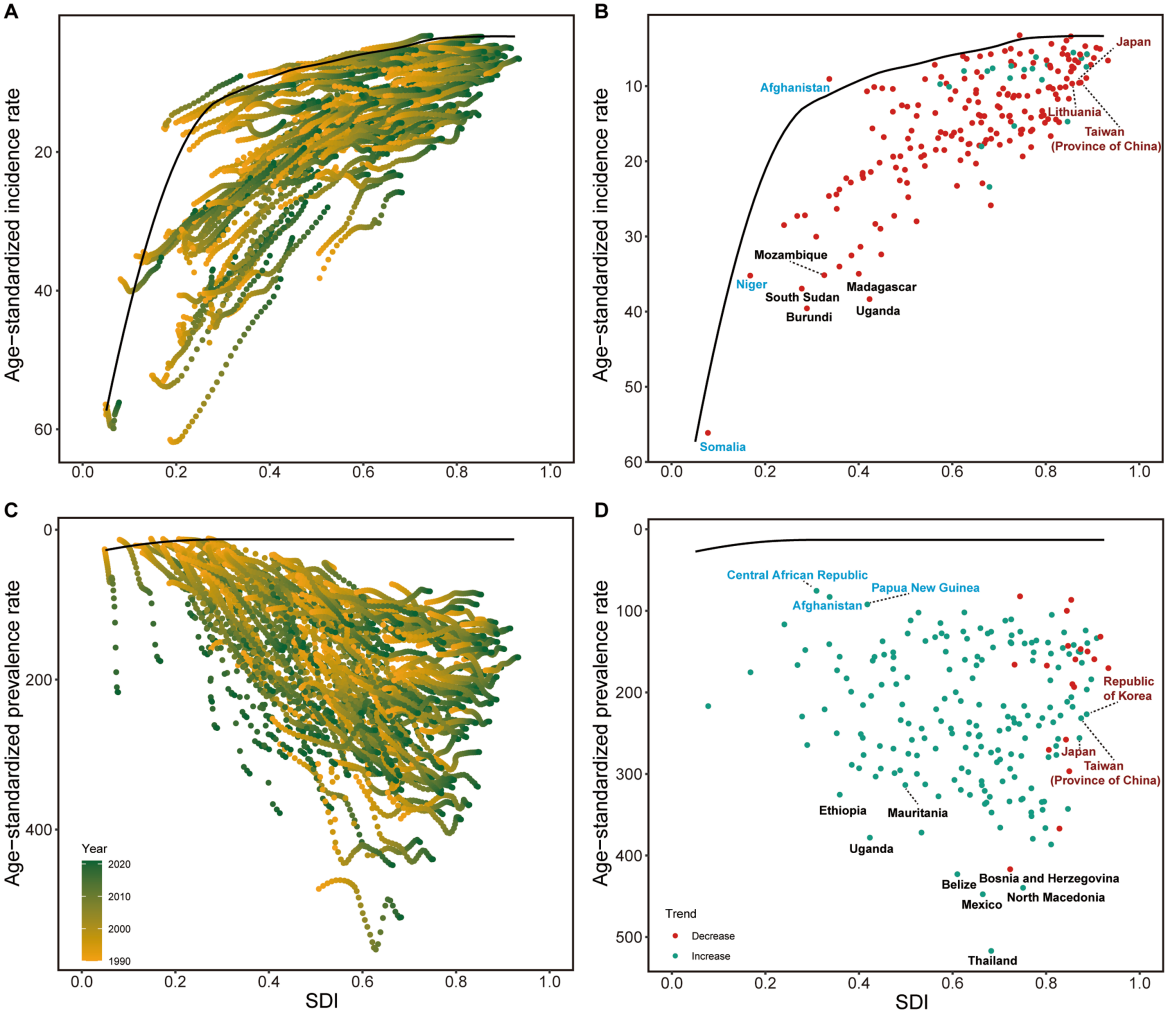
Frontier analysis of NE-BAT based on SDI and incidence and prevalence rate from 1990 to 2021. **(A)** Frontier analysis of age-standardized NE-BAT incidence rates from 1990 to 2021. **(B)** Frontier analysis of age-standardized NE-BAT incidence rates in 2021. **(C)** Frontier analysis of age-standardized NE-BAT prevalence rates from 1990 to 2021. **(D)** Frontier analysis of age-standardized NE-BAT prevalence rates in 2021. **(A,C)** Color scale represents the years from 1990 depicted in yellow to 2016 depicted in green. The frontier is delineated in solid black color. **(B,D)** The frontier is delineated in solid black color; countries and territories are represented as dots. Examples of frontier countries with low SDI (<0.5) and low effective difference are labeled in blue, and examples of countries and territories with high SDI (>0.85) and relatively high effective difference for their level of development are labeled in red. Red dots indicate an increase in age-standardized rate from 1990 to 2021; blue dots indicate a decrease in age-standardized rate from 1990 to 2021. NE-BAT, neonatal encephalopathy due to birth asphyxia and trauma; SDI, socio-demographic index.

Frontier analysis identified significant variations in country performance across all NE-BAT indicators, with notable differences both within and between SDI levels. The analysis revealed that between 1990 and 2021, incidence rates generally declined across most countries, irrespective of SDI level, with countries and territories at higher SDI levels showing convergence in performance (Figure 3A). Among low SDI countries and territories, Afghanistan, Niger, and Somalia performed best in reducing incidence rates, while Madagascar, Uganda, and Burundi were the worst performers. Conversely, among high SDI countries and territories, Japan, Lithuania, and Taiwan (Province of China) showed the poorest outcomes in incidence reduction (Figure 3B). In contrast to other indicators, prevalence rates showed a general increase from 1990 to 2021 (Figure 3C). For prevalence control, the Central African Republic, Afghanistan, and Papua New Guinea performed best among low SDI countries and territories, while Ethiopia, Uganda, and Mauritania were the worst performers. Among high SDI countries and territories, the Republic of Korea, Japan, and Taiwan (Province of China) demonstrated the poorest performance in controlling prevalence increases (Figure 3D).

**Figure 4:**
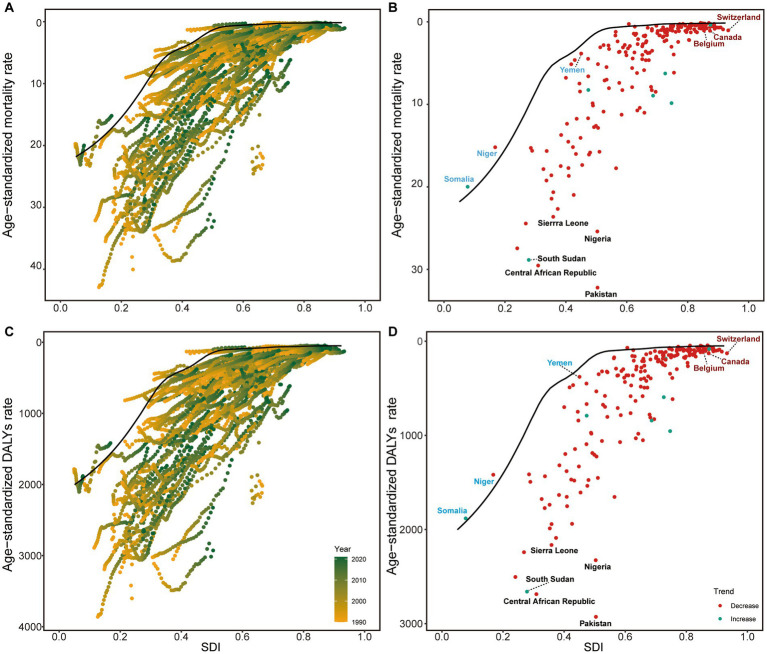
Frontier analysis of NE-BAT based on SDI and mortality and DALYs rate from 1990 to 2021. **(A)** Frontier analysis of age-standardized NE-BAT mortality rates from 1990 to 2021. **(B)** Frontier analysis of age-standardized NE-BAT mortality rates in 2021. **(C)** Frontier analysis of age-standardized NE-BAT DALYs rates from 1990 to 2021. **(D)** Frontier analysis of age-standardized NE-BAT DALYs rates in 2021. **(A,C)** Color scale represents the years from 1990 depicted in yellow to 2021 depicted in green. The frontier is delineated in solid black color. **(B,D)** The frontier is delineated in solid black color; countries and territories are represented as dots. Examples of frontier countries with low SDI (<0.5) and low effective difference are labeled in blue, and examples of countries and territories with high SDI (>0.85) and relatively high effective difference for their level of development are labeled in red. Red dots indicate an increase in age-standardized rate from 1990 to 2021; blue dots indicate a decrease in age-standardized rate from 1990 to 2021. NE-BAT, neonatal encephalopathy due to birth asphyxia and trauma; SDI, socio-demographic index; DALYs, disability-adjusted life years.

Mortality rates demonstrated similar patterns of general decline across SDI levels (Figure 4A). Somalia, Niger, and Yemen achieved the best performance in mortality reduction among low SDI countries and territories, while Canada, Belgium, and Switzerland showed the poorest outcomes among high SDI countries and territories (Figure 4B). DALYs patterns closely paralleled mortality trends (Figure 4C), with Niger, Yemen, and Somalia representing the best-performing low SDI countries and territories, and the same high SDI countries and territories (Canada, Belgium, and Switzerland) showing suboptimal performance (Figure 4D). These findings highlight both the potential for improvement in high-resource settings and the successful strategies implemented by certain low-resource countries that could serve as models for similar contexts.

### Projections of future burden

**Figure 5:**
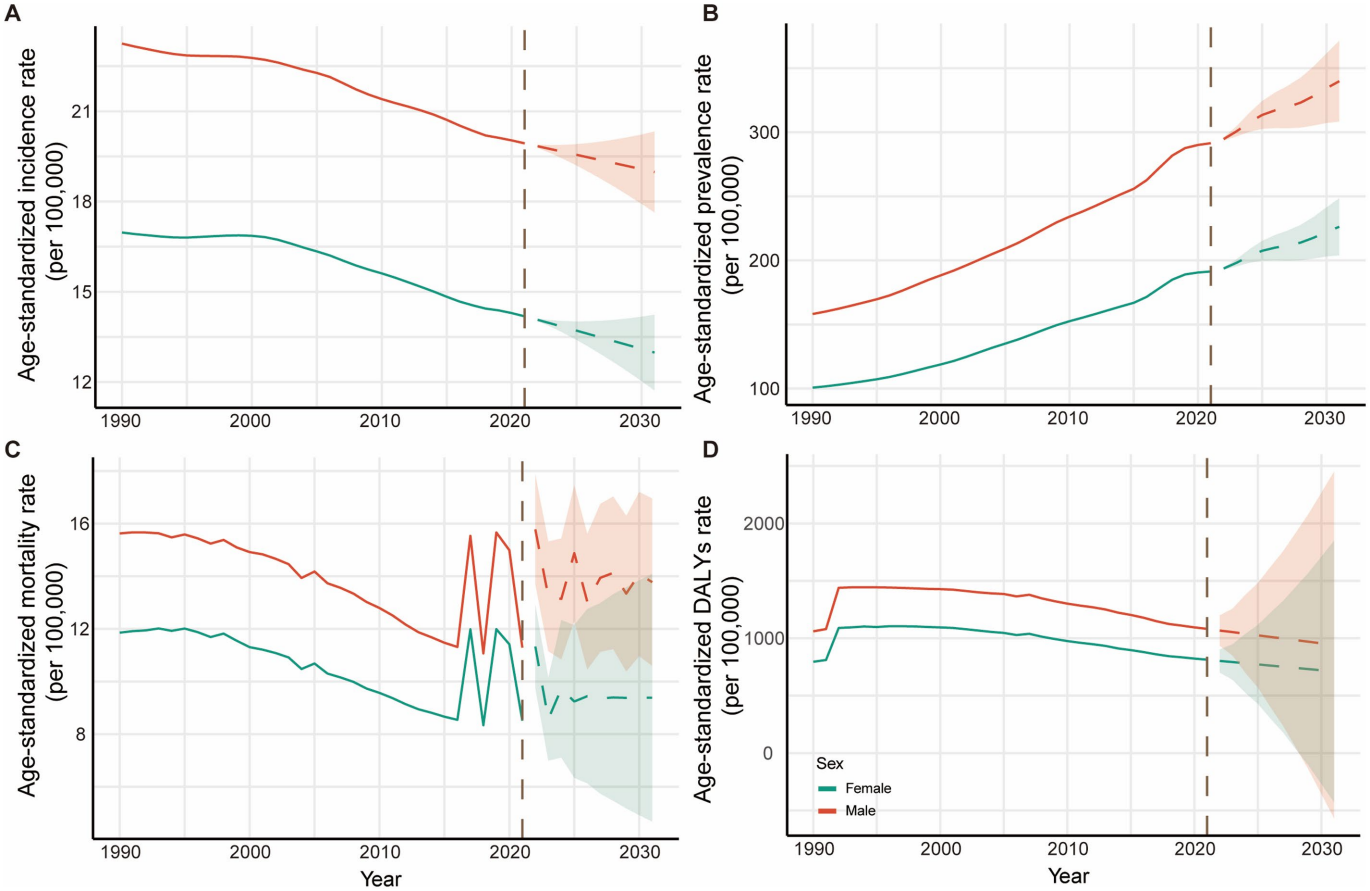
Trends and projection for NE-BAT by gender from 1990 to 2031. **(A)** Age-standardized incidence rates with historical trends (1990–2021) and future projections (2022–2031). **(B)** Age-standardized prevalence rates with historical trends and future projections. **(C)** Age-standardized mortality rates with historical trends and future projections. **(D)** Age-standardized DALYs rates with historical trends and future projections. The solid lines represent observed historical data (1990–2021), while dashed lines indicate ARIMA model projections (2022–2031). The vertical dashed line marks the transition from observed to projected data. Shaded areas represent 95% confidence intervals for projections. Red lines represent male; blue lines represent female. NE-BAT, neonatal encephalopathy due to birth asphyxia and trauma; DALYs, disability-adjusted life years.

ARIMA projections for NE-BAT burden from 2022 to 2031 are presented in Figure 5, with clear demarcation between historical observed data (1990–2021) and projected estimates indicated by the transition from solid to dashed line patterns. The projections reveal complex future trends across different indicators, with the shaded areas representing 95% confidence intervals reflecting the uncertainty inherent in these projections.

The incidence rates are projected to continue declining for both sexes, with males decreasing from 19.93 per 100,000 (95% UI: 19.67 to 20.24) in 2021 to 18.98 per 100,000 (95% CI: 17.63 to 20.32) by 2031, and females from 14.18 per 100,000 (95% UI: 13.88 to 14.51) to 12.98 per 100,000 (95% CI: 11.72 to 14.24) over the same period (Figure 5A; Supplementary Table S3). Conversely, prevalence rates show projected increases for both sexes, with males rising from 291.46 (95% UI: 253.38 to 330.70) per 100,000 to 339.83 per 100,000 (95% CI: 308.35 to 371.32) and females from 191.38 per 100,000 (95% UI: 166.48 to 216.29) to 226.25 per 100,000 (95% CI: 203.98 to 248.52) (Figure 5B; Supplementary Table S4), reflecting the survival paradox where improved acute care leads to increased numbers of survivors with long-term disabilities. Mortality rates demonstrate a concerning projected reversal of the historical declining trend, with males projected to rise from 11.29 per 100,000 (95% UI: 9.37 to 13.36) to 13.78 per 100,000 (95% CI: 10.60 to 16.95) and females from 8.51 per 100,000 (95% UI: 7.13 to 10.06) to 9.38 per 100,000 (95% CI: 4.67 to 14.09) (Figure 5C; Supplementary Table S5). DALYs rates are projected to continue their declining trend, from 1,081.96 per 100,000 (95% UI: 911.78 to 1257.95) in 2021 to 941.66 per 100,000 (95% CI: −568.23 to 2451.55) by 2031 for males and from 813.53 per 100,000 (95% UI: 697.46 to 951.16) to 710.76 per 100,000 (95% UI: −427.82 to 1849.35) for females (Figure 5D; Supplementary Table S6).

## Discussion

This study provides a comprehensive analysis of global NE-BAT trends from 1990 to 2021, revealing complex patterns in disease burden and highlighting both significant progress and persistent challenges in addressing NE-BAT globally. While NE-BAT represents a specific subset of neonatal encephalopathy, it is important to acknowledge that neonatal encephalopathy encompasses a multifactorial etiology including hypoxic–ischemic encephalopathy, infection, intracranial hemorrhage, stroke, brain malformations, metabolic disorders, and genetic causes, each requiring specific diagnostic approaches and management strategies (16).

The observed annual decrease in global ASIR coupled with substantial increase in prevalence reflects the profound impact of medical advances on NE-BAT outcomes over the past three decades. This apparent paradox stems from simultaneous improvements in both prevention and treatment capabilities. Enhanced prenatal care and accessible neonatal resuscitation strategies have reduced the incidence of new cases, aligning with previous studies documenting reduced birth asphyxia rates (17), while revolutionary advances in acute care management - particularly the establishment of therapeutic hypothermia as standard treatment and the development of comprehensive diagnostic approaches including amplitude-integrated electroencephalography (aEEG), video EEG monitoring and advanced neuroimaging - have dramatically improved survival rates (16, 18–20).

Contemporary advances in obstetric monitoring, including continuous fetal heart rate monitoring and improved cesarean delivery protocols, have substantially reduced severe birth asphyxia events (21). Furthermore, the widespread implementation of therapeutic hypothermia has resulted in a transformation of survival trajectories for affected neonates (22). However, this medical progress has created a “survival paradox” where these neuroprotective interventions significantly improve immediate survival rates but generate a growing population of children requiring comprehensive long-term neurodevelopmental support services (23, 24). This evolving pattern necessitates expanded healthcare resources for managing neurodevelopmental disorders, rehabilitation, and family support, particularly challenging low-resource settings where such services remain limited.

While these advances have benefited all populations, our analysis reveals persistent disparities that require targeted attention, particularly the consistently higher burden observed among male neonates. Our findings of consistently higher male incidence and prevalence rates align with recent systematic evidence demonstrating increased male vulnerability to perinatal complications. Meta-analytical evidence spanning 20 years reveals significant female advantages in neurodevelopmental outcomes among premature infants (25), supported by comprehensive reviews documenting sex differences in neonatal brain injury and inflammation, which confirm that males face disproportionate risks with distinct inflammatory response patterns and increased microglial activation (26). The neurobiological basis involves estrogen receptor-*α*-mediated neuroprotection in females (27) and X-chromosome-linked protective factors and sexually dimorphic inflammatory responses, which can influence the severity of birth asphyxia and trauma (28, 29). These findings are further corroborated by recent high-quality primary research, including a 2024 cross-sectional study of 196 neonates that rigorously demonstrated gender-specific associations of multiple risk factors with moderate or severe hypoxic–ischemic encephalopathy (30). This gender-specific vulnerability warrants consideration in risk assessment and intervention strategies, as males may require more aggressive early interventions to mitigate long-term effects.

Beyond individual biological factors, broader healthcare system characteristics also drive NE-BAT burden variations globally. The marked disparities between high and low SDI locations (27-fold difference in mortality rates) underscore the critical role of healthcare infrastructure and resources (31). While the dramatic disparities between low and high SDI locations capture attention, the patterns observed in middle- and high-income countries reveal important nuances in global progress against NE-BAT. Middle-income countries demonstrated the most variable improvement trajectories, with some achieving mortality reductions that exceeded those seen in high-income settings, particularly among the high-middle SDI category. These variations among countries with similar SDI levels highlight that specific healthcare capabilities and clinical practices may be more influential than socioeconomic factors alone. International surveys also reveal significant practice variations across regions, including differences in therapeutic hypothermia eligibility criteria, HIE severity grading systems, and access to advanced neuromonitoring techniques (32). The expanding application of therapeutic hypothermia to mild HIE cases, with 94.5% of mild HIE cases now receiving treatment despite limited evidence, demonstrates how practice patterns evolve differently across healthcare systems (33). Additionally, variations in neuroimaging timing and interpretation, neurodevelopmental follow-up protocols, and the integration of advanced techniques such as amplitude-integrated electroencephalography and MR spectroscopy contribute to outcome disparities (32, 34–36). Recent advances in combining hypothermia with neuroprotective adjuvants have shown promise in reducing hospitalization duration, though access to such combined therapies varies significantly across different healthcare settings (37). High-income countries, despite maintaining the lowest absolute mortality rates throughout the study period, showed more modest percentage improvements, reflecting the inherent challenges of achieving further gains when baseline performance is already optimized. Low SDI locations face multiple challenges, including limited access to skilled birth attendants, inadequate monitoring equipment, and insufficient neonatal intensive care facilities, all of which contribute to higher mortality rates and poorer outcomes (38–40). The success observed in some middle-income countries offers valuable lessons for other regions with similar resource constraints, particularly regarding cost-effective implementation of evidence-based protocols and strategic resource allocation for maximum impact.

Healthcare system capacity and clinical practice variations may explain these disparities. The effectiveness of evidence-based interventions varies significantly across settings due to differences in resource availability and training infrastructure (17, 41). Even basic resuscitation training can substantially improve survival outcomes, while healthcare delivery variations affect both actual burden and estimation accuracy (6, 19, 42). The slower decline in incidence rates in high SDI locations (−0.35 vs. -0.87 in low SDI) might reflect a “floor effect,” where further improvements become increasingly challenging once basic preventive measures have been implemented (43). Additionally, data quality and reporting systems vary considerably between countries, with some locations having limited capacity for accurate case identification and reporting, potentially affecting apparent burden estimates (44, 45). These multifaceted factors underscore the importance of addressing country-specific healthcare system strengthening beyond economic development indicators to achieve effective NE-BAT burden reduction.

Despite these challenges, the improvements observed in Eastern Europe, East Asia, and Central Europe demonstrate the potential for rapid progress through systematic healthcare reforms. Countries like Somalia, Niger, and Yemen, despite resource limitations, have shown remarkable improvements in mortality reduction, suggesting the effectiveness of targeted interventions even in challenging settings. Their success may offer valuable lessons for other low-resource regions, where improvements in neonatal care are achievable through tailored strategies that address local healthcare gaps, such as training community health workers, improving access to essential equipment, and implementing cost-effective resuscitation protocols (6).

The ARIMA projections indicating continued decline in incidence but rising prevalence and mortality rates by 2031 present complex policy implications. These projections align with economic evaluation studies emphasizing the importance of quantitative disease burden estimates for resource allocation and public health prioritization (6). The projected increase in mortality rates may reflect the growing complexity of cases surviving the acute phase. Strategic planning must address both immediate prevention needs and sustainable healthcare system capacity to manage evolving burden patterns (42, 45, 46).

### Limitations and future directions

While this study provides valuable insights into the global burden of NE-BAT, there are several limitations. The reliance on GBD estimates introduces inherent uncertainties related to data quality variations across countries, particularly in regions with limited health information systems, disrupted civil registration systems or political instability. Countries such as Afghanistan and Yemen - which are highlighted in our analysis - often have particularly uncertain data quality due to ongoing conflicts and compromised health surveillance systems. Our ARIMA projections, while methodologically robust as demonstrated in recent epidemiological forecasting studies (15), assume continuation of historical trends and cannot predict the impact of future policy interventions, technological breakthroughs or unexpected events such as pandemics, political conflicts or natural disasters that could substantially alter NE-BAT burden trajectories. Future studies could benefit from incorporating more granular data from national and local health surveys to better capture regional variations and healthcare system differences. Additionally, while the study highlights the increasing prevalence of disabilities, it does not provide in-depth analysis of the specific types of neurodevelopmental outcomes associated with NE-BAT, such as cerebral palsy, cognitive impairments, or epilepsy. Further research is needed to explore these outcomes and their long-term impact on quality of life.

Looking ahead, the projection models suggest that, while mortality and DALYs are expected to decline, the rising prevalence of NE-BAT-related disabilities presents a growing challenge. Policymakers should focus on strengthening both preventive measures during childbirth and long-term care for affected infants, particularly in low-resource settings. Increased attention should also be paid to gender-specific interventions, given the higher incidence and prevalence rates among males. Future research should aim to identify effective interventions that can mitigate the impact of NE-BAT, reduce regional disparities, and improve the quality of life for survivors.

## Conclusion

This study reveals the evolving complexity of NE-BAT burden globally. While substantial progress has been achieved in reducing incidence and mortality, the increasing prevalence of long-term disabilities presents new challenges for healthcare systems. These findings support the implementation of differentiated intervention strategies aligned with healthcare system capabilities and documented burden patterns. Low SDI countries should prioritize high-impact interventions including systematic skilled birth attendant training programs and establishment of functional obstetric emergency referral networks. Middle-income countries should focus on therapeutic hypothermia implementation combined with comprehensive neurodevelopmental follow-up services, leveraging their expanding healthcare infrastructure. High-income countries should emphasize development of long-term disability support systems and precision medicine approaches that optimize individualized care protocols. The consistent gender disparities observed across all settings necessitate integration of sex-specific risk assessment protocols and enhanced monitoring strategies for male neonates. Healthcare systems must also prepare for the projected increase in survivor populations requiring comprehensive long-term care services, including early intervention programs and family support networks. The persistent regional disparities underscore the need for context-specific interventions that address both immediate prevention needs and long-term care requirements. Future research should focus on developing effective strategies to optimize outcomes across the complete spectrum of NE-BAT care - from prevention through long-term neurodevelopmental support.

## Data Availability

The original contributions presented in the study are included in the article/Supplementary material, further inquiries can be directed to the corresponding author/s.
