# Supplementary material for: Neonatal encephalopathy due to birth asphyxia and trauma: global trends and disparities
Source: Front Public Health. 2025 Sep 11;13:1583572. doi: 10.3389/fpubh.2025.1583572 (PMC12460252; doi:10.3389/fpubh.2025.1583572)
Supplement: Supplementary file 1 [file Data_Sheet_1.pdf]

Supplementary Material

1 Supplementary Methods

1.1 Overview

The Global Burden of Disease (GBD) 2021 study provides comprehensive estimates for 204 countries and territories, organized into 21 regions and 7 super-regions. The study estimates incidence, prevalence, years lived with disability (YLDs), and disability-adjusted life years (DALYs) from 1990 to 2021, as well as deaths and years of life lost (YLLs) from 1980 to 2021. The GBD cause list is hierarchically organized, with neonatal encephalopathy due to birth asphyxia and trauma (NE-BAT) classified within the broader category of neonatal disorders. Results are accessible through the Global Health Data Exchange (GHDx), which provides interactive tools for data access and download.

1.2 Case definitions

Neonatal encephalopathy due to birth asphyxia and trauma (NE-BAT) is defined according to GBD classification as neonatal encephalopathy resulting from oxygen deprivation (asphyxia) and/or mechanical injury (trauma) during the perinatal period, as distinguished from other causes of neonatal encephalopathy such as genetic, metabolic, or infectious etiologies. This classification aligns with ICD-10 codes P02-P03.9 (fetus and newborn affected by complications of placenta, cord and membranes), P10-P15.9 (birth trauma), P20-P21.9 (intrauterine hypoxia and birth asphyxia), P24-P24.9 (neonatal aspiration), P52-P52.9 (intracranial nontraumatic hemorrhage of newborn), and P90-P91.9 (other disorders of cerebral status of newborn), representing a specific subset of neonatal encephalopathy with distinct pathophysiological mechanisms and prevention strategies.

1.3 Term definitions

Incidence: The number of new cases of NE-BAT that occur in a specified population within a defined time period, measuring the rate at which new cases develop.

Prevalence: The total number of cases, both new and pre-existing, of NE-BAT in a specified population at a given time. GBD models prevalence across multiple age groups including birth, early neonatal (0-6 days), late neonatal (7-27 days), and extends to post-neonatal periods (<28 days, 1-5 months, 6-11 months, 12-23 months, 2-4 years) to capture the long-term burden of survivors with disabilities.

Years of Life Lost (YLLs): The total number of years lost due to premature death from NE-BAT, calculated by subtracting the age at death from standard life expectancy.

Years Lived with Disability (YLDs): Years of healthy life lost due to living with NE-BAT-related disabilities, calculated by multiplying prevalence by disability weights reflecting condition severity. Disability weights for NE-BAT sequelae include motor impairments (mild: 0.01, moderate: 0.061, severe: 0.402), cognitive impairments, epilepsy, and blindness.

Disability-Adjusted Life Years (DALYs): A summary measure combining YLLs and YLDs, representing total years lost due to both premature death and disability from NE-BAT.

Socio-demographic Index (SDI): A composite measure assessing development status based on income per capita, average years of schooling, and total fertility rate under age 25, ranging from 0 to 1.

1.4 Input data

For GBD 2021, NE-BAT data were sourced from vital registration systems, hospital records, and survey data. Data quality varied significantly across regions, with high-income countries providing more complete vital registration data, while low- and middle-income countries relied more heavily on survey estimates and modeled data. The GBD modeling framework addressed data gaps through statistical imputation methods and uncertainty propagation techniques.

Birth asphyxia cases were identified through clinical criteria including low Apgar scores, need for resuscitation, and evidence of multi-organ dysfunction. Birth trauma cases included mechanical injuries during delivery such as intracranial hemorrhage, nerve injuries, and skeletal fractures. Data adjustments were applied using Bayesian meta-regression tools (MR-BRT) to harmonize different case definitions and diagnostic criteria across data sources.

1.5 Modeling

The GBD 2021 NE-BAT estimates employed a comprehensive six-step modeling approach: Step 1: Model NE prevalence at birth using spatiotemporal Gaussian process regression (ST-GPR). Step 2: Estimate NE prevalence in early neonatal, late neonatal, and 28-day periods using life table algorithm. Step 3: Model case fatality ratio and impairment proportions (asymptomatic, mild, moderate-severe) at 28 days using mixed-effects regressions. Step 4: Model long-term impairment prevalence at all ages based on 28-day prevalence. Step 5: Split mild and moderate/severe impairment prevalence into specific sequelae. Step 6: Apply disability weights to calculate years lived with disability (YLDs).

Uncertainty Quantification: The GBD framework generated 1000 draws at each computational step, propagating uncertainty from multiple sources: Input data availability and quality variations; Model structure and parameter estimation uncertainty; Corrections for measurement error and bias adjustments; Geographic and temporal modeling uncertainties.

The 95% uncertainty intervals (UIs) represent the 25th and 975th values of these ordered draws, following established GBD methodology for comprehensive uncertainty propagation.

1.6 Statistical Analysis

Unlike previous descriptive GBD analyses, our study employs an integrated analytical framework combining temporal trend analysis, frontier performance evaluation and predictive modeling to provide comprehensive insights for healthcare planning and policy development.

Trend Analysis: Estimated Annual Percentage Change (EAPC) was calculated to quantify temporal trends in age-standardized rates from 1990 to 2021. EAPC was derived by fitting linear regression models to the natural logarithm of rates over time, with the formula: EAPC = $(e^\beta - 1) \times 100\%$, where β represents the regression coefficient.

Frontier Analysis: A frontier analysis was conducted to assess the performance of countries and territories in reducing NE-BAT burden, adjusting for SDI levels. To improve robustness, 1000 bootstrap samples were generated, with each sample drawn with replacement. For each iteration, the

frontier was constructed by calculating the cumulative minimum of ASIR, ASPR, ASMR and ASDR after ordering by SDI. The final frontier curve was generated by averaging the bootstrapped frontiers, and locally weighted regression (LOESS) smoothing was applied to reduce the impact of extreme values.

LOESS Implementation: The LOESS algorithm used a smoothing parameter (span) of 0.75, selected through cross-validation to balance smoothness with local responsiveness. The method creates non-parametric smooth curves without assuming specific functional forms, making it ideal for identifying optimal performance boundaries across varying development levels. Each country's actual NE-BAT burden was compared to the smoothed frontier curve, providing measures of relative performance and identifying top-performing countries.

ARIMA Modeling for Projections: Autoregressive Integrated Moving Average (ARIMA) models were employed to forecast NE-BAT burden from 2022 to 2031. The modeling process involved: Data Preprocessing: Age-standardized rates were log-transformed to achieve stationarity and normalize residuals. Model Selection: ARIMA parameters (p, d, q) were selected using the Akaike Information Criterion (AIC) and Bayesian Information Criterion (BIC), with automated selection procedures implemented in R. Stationarity Testing: Augmented Dickey-Fuller tests were performed to determine the degree of differencing required. Model Diagnostics: Ljung-Box tests assessed residual autocorrelation, and Jarque-Bera tests evaluated normality assumptions. Forecasting: Models were fitted to 1990-2021 data and used to generate 10-year projections with 95% confidence intervals.

Model Specifications: Separate ARIMA models were fitted for incidence, prevalence, mortality, and DALYs rates, stratified by sex. The final models incorporated seasonal adjustments where appropriate and included drift terms to capture long-term trends.

2 Supplementary Figures

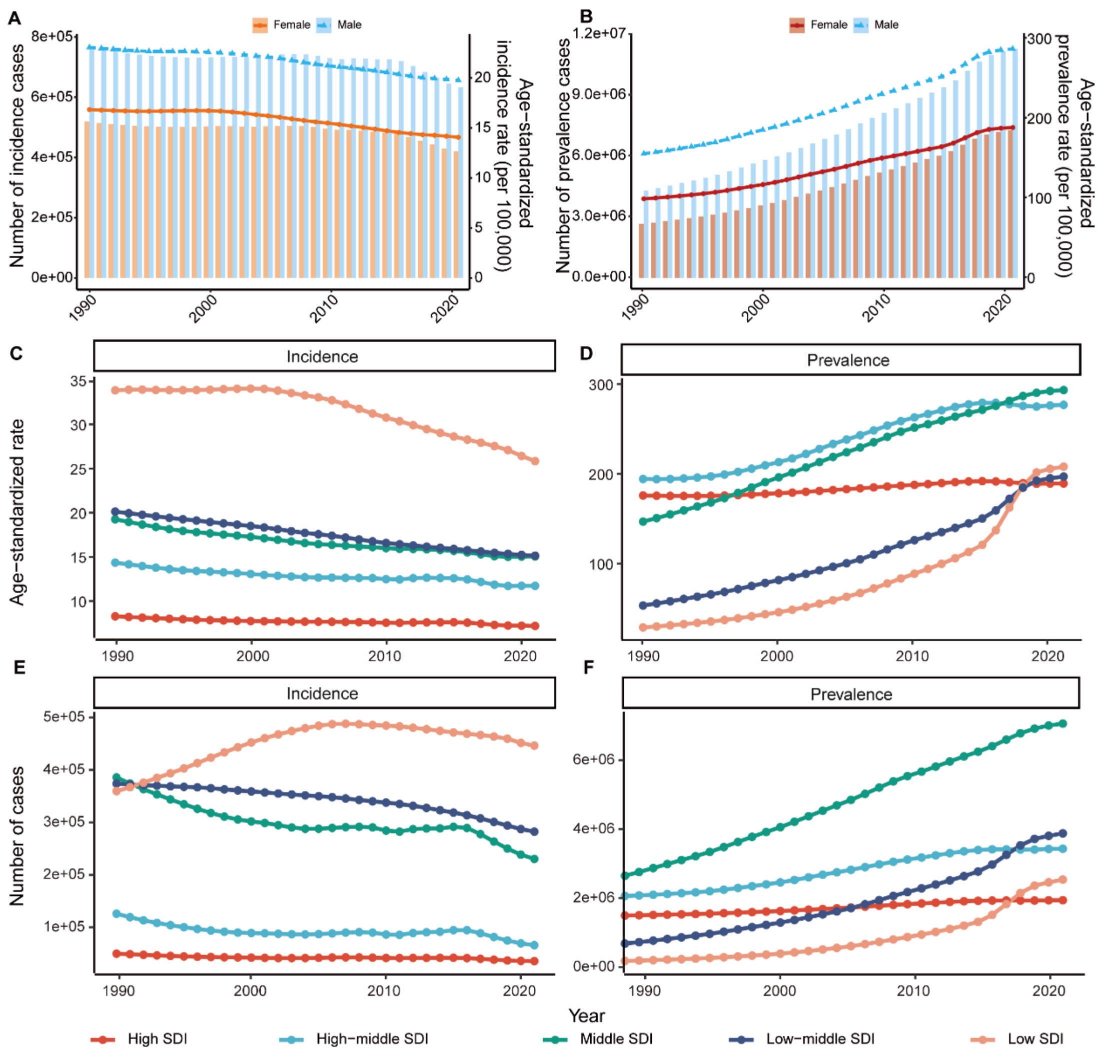

Figure S1. Trends in Burden of NE-BAT Grouped by Sexes and SDI Quintiles from 1990 to 2021. A-B The number of cases and the age-standardized rates for incidence (**A**) and prevalence (**B**) of NE-BAT from 1990 to 2021. **C-D** The age-standardized rate of incidence and prevalence due to NE-BAT grouped by SDI quintiles from 1990 to 2021. **E-F** The number of cases for incidence and prevalence due to NE-BAT grouped by SDI quintiles from 1990 to 2021. *NE-BAT* neonatal encephalopathy due to birth asphyxia and trauma, *SDI* Socio-demographic index.

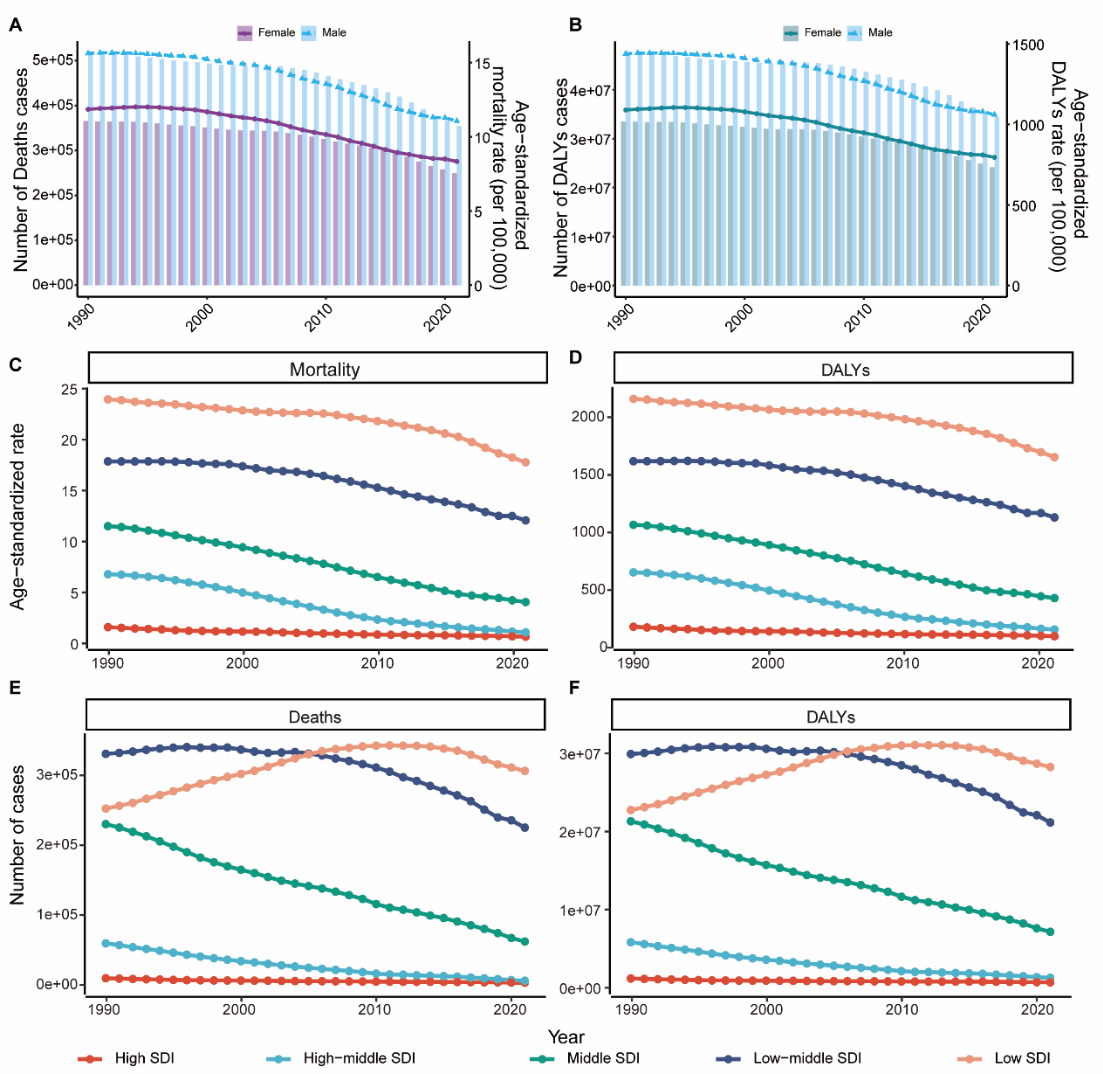

Figure S2. Trends of Mortality and DALYs of NE-BAT Grouped by Sexes and SDI Quintiles from 1990 to 2021. A-B The number of cases and the age-standardized rates for mortality (**A**) and DALYs (**B**) of NE-BAT from 1990 to 2021. **C-D** The age-standardized rate of mortality and DALYs due to NE-BAT grouped by SDI quintiles from 1990 to 2021. **E-F** The number of cases for deaths and DALYs due to NE-BAT grouped by SDI quintiles from 1990 to 2021. *DALYs* disability-adjusted life years, *NE-BAT* neonatal encephalopathy due to birth asphyxia and trauma. *SDI* Socio-demographic index.

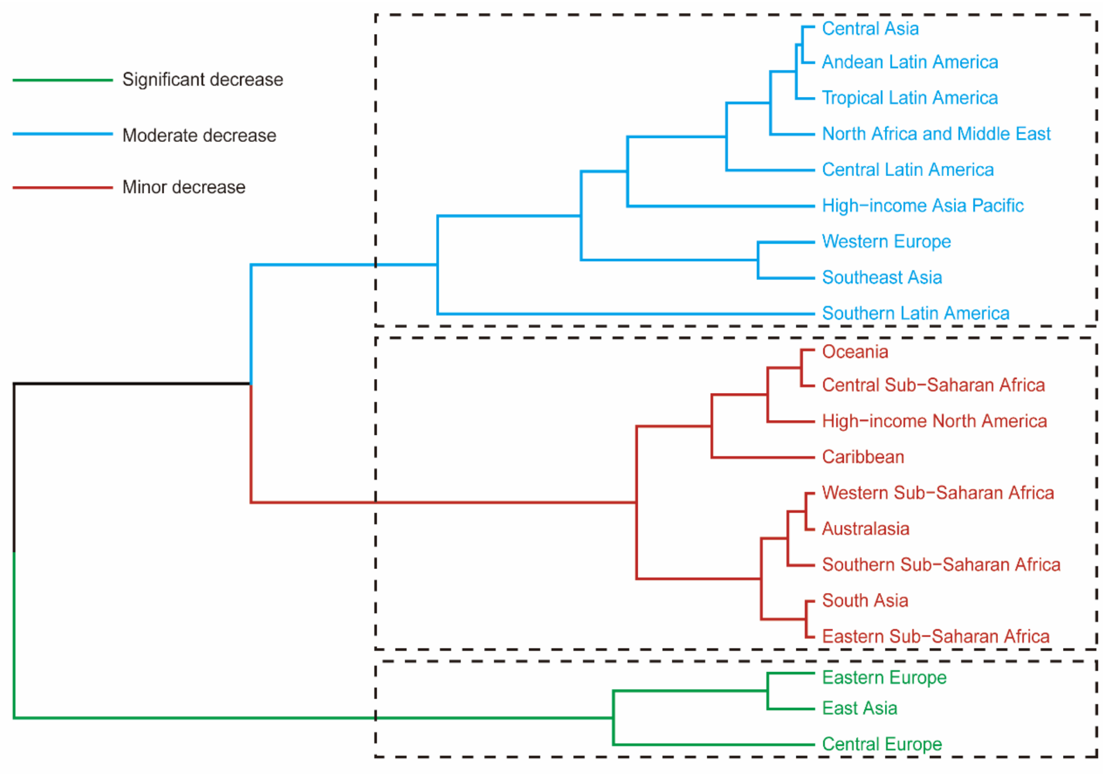

Figure S3. Results of Cluster Analysis Based on the EAPC Values of NE-BAT related ASMR and ASDR from 1990 to 2021. *EAPC* estimated annual percentage change, *NE-BAT* neonatal encephalopathy due to birth asphyxia and trauma, *ASMR* Age-standardized mortality rates, *ASDR* Age-standardized DALYs rates, *DALYs* disability-adjusted-life-years.
